# Supplementary material for: VinDr-CXR: An open dataset of chest X-rays with radiologist’s annotations
Source: Sci Data. 2022 Jul 20;9:429. doi: 10.1038/s41597-022-01498-w (PMC9300612; doi:10.1038/s41597-022-01498-w)
Supplement: Supplementary file 1 — inDr-CXR: An open dataset of chest X-rays with radiologist's annotations (Supplementary materials) [file 41597_2022_1498_MOESM1_ESM.pdf]

VinDr-CXR: An open dataset of chest X-rays with radiologist's annotations (Supplementary materials)

Contents

1 List of DICOM tags 2
2 Definition of findings and diseases 3
3 Examples of disagreements between radiologists 4

1 List of DICOM tags

Table 1. The list of DICOM tags that were retained for loading and processing raw images. All other tags were removed for protecting patient privacy. Details about all these tags can be found from DICOM Standard Browser at https://dicom.innolitics.com/ciods.

DICOM Tag	Attribute Name	Description
(0010, 0040)	Patient's Sex	Sex of the named patient.
(0010, 1010)	Patient's Age	Age of the patient.
(0010, 1020)	Patient's Size	Length or size of the patient, in meters.
(0010, 1030)	Patient's Weight	Weight of the patient, in kilograms.
(0028, 0010)	Rows	Number of rows in the image.
(0028, 0011)	Columns	Number of columns in the image.
(0028, 0030)	Pixel Spacing	Physical distance in the patient between the center of each pixel, specified by a numeric pair - adjacent row spacing (delimiter) adjacent column spacing in mm.
(0028, 0034)	Pixel Aspect Ratio	Ratio of the vertical size and horizontal size of the pixels in the image specified by a pair of integer values where the first value is the vertical pixel size, and the second value is the horizontal pixel size.
(0028, 0100)	Bits Allocated	Number of bits allocated for each pixel sample. Each sample shall have the same number of bits allocated.
(0028, 0101)	Bits Stored	Number of bits stored for each pixel sample. Each sample shall have the same number of bits stored.
(0028, 0102)	High Bit	Most significant bit for pixel sample data. Each sample shall have the same high bit.
(0028, 0103)	Pixel Representation	Data representation of the pixel samples. Each sample shall have the same pixel representation.
(0028, 0106)	Smallest Image Pixel Value	The minimum actual pixel value encountered in this image.
(0028, 0107)	Largest Image Pixel Value	The maximum actual pixel value encountered in this image.
(0028, 1050)	Window Center	Window center for display.
(0028, 1051)	Window Width	Window width for display.
(0028, 1052)	Rescale Intercept	The value b in relationship between stored values (SV) and the output units specified in Rescale Type (0028,1054). Each output unit is equal to m*SV + b.
(0028, 1053)	Rescale Slope	Value of m in the equation specified by Rescale Intercept (0028,1052).
(7FE0, 0010)	Pixel Data	A data stream of the pixel samples that comprise the image.
(0028, 0004)	Photometric Interpretation	Specifies the intended interpretation of the pixel data.
(0028, 2110)	Lossy Image Compression	Specifies whether an image has undergone lossy compression (at a point in its lifetime).
(0028, 2114)	Lossy Image Compression Method	A label for the lossy compression method(s) that have been applied to this image.
(0028, 2112)	Image Compression Ratio	Describes the approximate lossy compression ratio(s) that have been applied to this image.
(0028, 0002)	Samples per Pixel	Number of samples (planes) in this image.
(0028, 0008)	Number of Frames	Number of frames in a multi-frame image.

2 Definition of findings and diseases

Table 2. Definition of findings and diseases used in the study.

	Pathology label	Definition
Local Label	1. Aortic enlargement	An abnormal bulge that occurs in the wall of the major blood vessel.
	2. Atelectasis	Collapse of a part of the lung due to a decrease in the amount of air in the alveoli resulting in volume loss and increased density.
	3. Cardiomegaly	Enlargement of the heart, occurs when the heart of an adult patient is larger than normal and the cardiothoracic ratio is greater than 0.5.
	4. Calcification	Deposition of calcium salts in the lung.
	5. Clavicle fracture	A break in the collarbone.
	6. Consolidation	Any pathologic process that fills the alveoli with fluid, pus, blood, cells (including tumor cells) or other substances resulting in lobar, diffuse or multifocal ill-defined opacities.
	7. Edema	Fluid accumulation in the tissur and air space of the lungs.
	8. Emphysema	A condition of the lung characterized by an abnormal increase in the size of air spaces distal to the terminal bronchioles.
	9. Enlarged PA	Dilatation of the pulmonary artery - a defect characterized by a wider than normal main pulmonary artery.
	10. Interstitial lung disease (ILD)	Involvement of the supporting tissue of the lung parenchyma resulting in fine or coarse reticular opacities or small nodules.
	11. Infiltration	An abnormal substance that accumulates gradually within cells or body tissues or any substance or type of cell that occurs within or spreads as through the interstices (interstitium and/or alveoli) of the lung, that is foreign to the lung, or that accumulates in greater than normal quantity within it.
	12. Lung cavity	Thick-walled abnormal gas-filled spaces within the lung. They are usually associated with a nodule, mass, or area of consolidation. A fluid level within the space may be present.
	13. Lung cyst	Lung cysts refer to round, thin-walled, low attenuation spaces/lucencies in the lung.
	14. Lung opacity	Any abnormal focal or generalized opacity or opacities in lung fields (blanket tag including but not limited to consolidation, cavity, fibrosis, nodule, mass, calcification, interstitial thickening, etc.).
	15. Mediastinal shift	The deviation of the mediastinal structures towards one side of the chest cavity.
	16. Nodule/Mass	Any space occupying lesion either solitary or multiple.
	17. Pulmonary fibrosis	An excess of fibrotic tissue in the lung.
	18. Pneumothorax	The presence of gas (air) in the pleural space.
	19. Pleural thickening	Any form of thickening involving either the parietal or visceral pleura.
	20. Pleural effusion	Abnormal accumulations of fluid within the pleural space.
	21. Rib fracture	A common injury that occurs when one of the bones in your rib cage breaks or cracks.
	22. Other lesion	Other lesions that are not on the list of findings or abnormalities mentioned above.
Global labels	23. Lung tumor	The result of abnormal rates of cell division or cell death in lung tissue, or in the airways that lead to the lungs.
	24. Pneumonia	An infection that inflames the air sacs in one or both lungs.
	25. Tuberculosis	Any sign suggesting pulmonary or extrapulmonary tuberculosis.
	26. Other diseases	Other diseases that are not on the list of diseases mentioned above.
	27. COPD	Chronic obstructive pulmonary disease (COPD) is defined as a condition characterized by persistent airflow limitation that is usually progressive and associated with an enhanced chronic inflammatory response in the airways and the lung to noxious particles or gases.
	28. No finding	The absence of all pathologies from the chest radiograph.

3 Examples of disagreements between radiologists

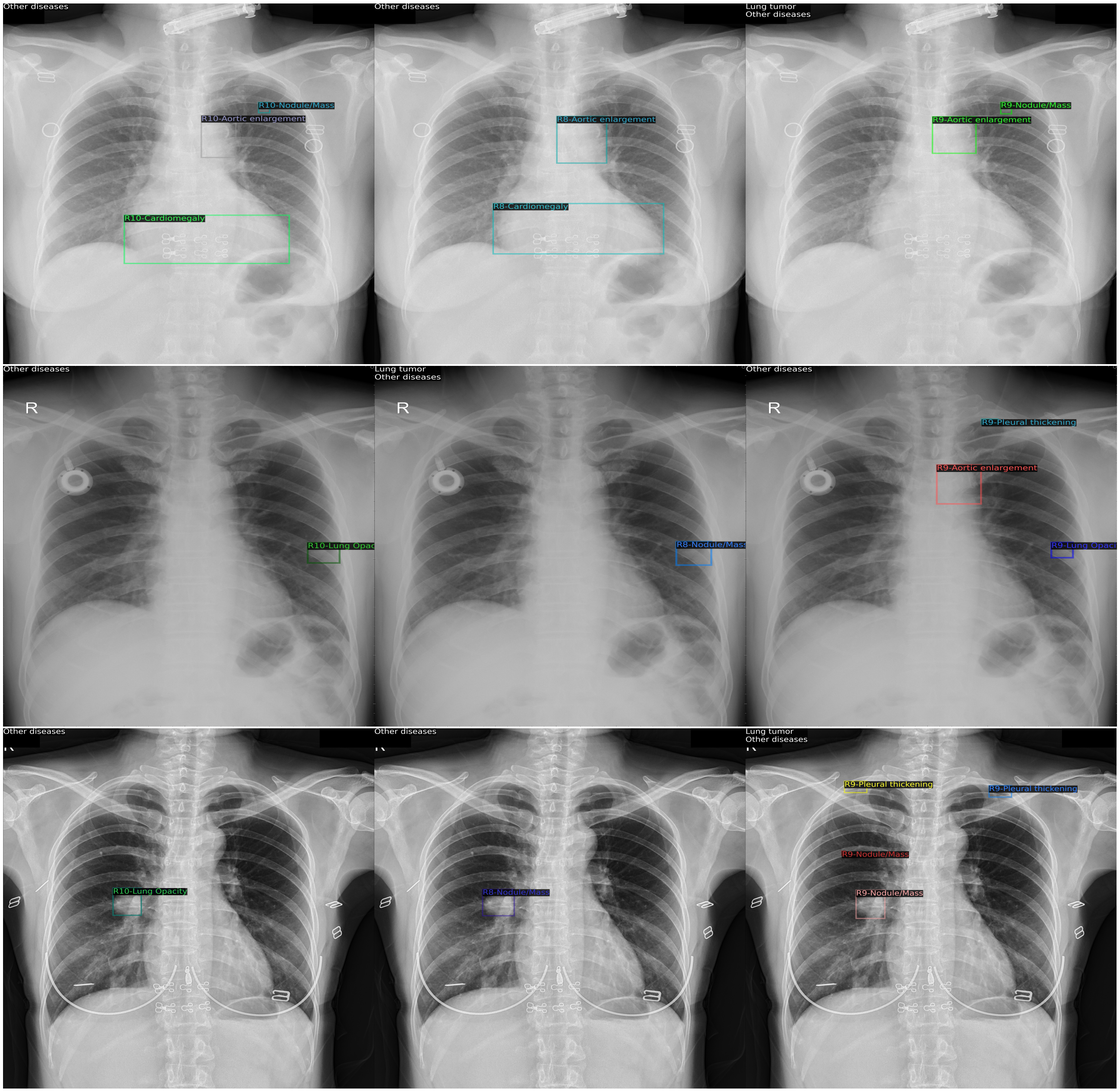

Figure 1. Examples of disagreements between radiologists. Abnormal findings are marked by radiologists via rectangle bounding boxes. R8, R9, R10 are referred to three independent radiologists.
